# The Ohio State Journal of Dental Science

**Published:** 1881-05

**Authors:** 


*The Ohio State Journal of Dental Science.* Edited by George Watt, M. D., D. D. S., Xenia, Ohio.

We gladly welcome this new addition to dental literature, and commend the first two numbers received as worthy of their editor, who has long occupied a prominent position in the dental profession. The manner in which the new Journal is gotten up, and its appearance are very creditable, and we trust it may not only prove a permanent but a successful publication. The terms are $1.50 per year in advance, the subscription price being an evidence of the desire of its author to make it a labor of love for his profession.

